# Dynamic Replacement of Histone H3 Variants Reprograms Epigenetic Marks in Early Mouse Embryos

**DOI:** 10.1371/journal.pgen.1002279

**Published:** 2011-10-06

**Authors:** Tomohiko Akiyama, Osamu Suzuki, Junichiro Matsuda, Fugaku Aoki

**Affiliations:** 1 Department of Integrated Biosciences, Graduate School of Frontier Sciences, University of Tokyo, Kashiwa, Japan; 2 Laboratory of Animal Models for Human Diseases, National Institute of Biomedical Innovation, Ibaraki, Japan; Medical Research Council Human Genetics Unit, United Kingdom

## Abstract

Upon fertilization, reprogramming of gene expression is required for embryo development. This step is marked by DNA demethylation and changes in histone variant composition. However, little is known about the molecular mechanisms causing these changes and their impact on histone modifications. We examined the global deposition of the DNA replication-dependent histone H3.1 and H3.2 variants and the DNA replication-independent H3.3 variant after fertilization in mice. We showed that H3.3, a euchromatic marker of gene activity, transiently disappears from the maternal genome, suggesting erasure of the oocyte-specific modifications carried by H3.3. After fertilization, H3.2 is incorporated into the transcriptionally silent heterochromatin, whereas H3.1 and H3.3 occupy unusual heterochromatic and euchromatin locations, respectively. After the two-cell stage, H3.1 and H3.3 variants resume their usual respective locations on heterochromatin and euchromatin. Preventing the incorporation of H3.1 and H3.2 by knockdown of the histone chaperone CAF-1 induces a reciprocal increase in H3.3 deposition and impairs heterochromatin formation. We propose that the deposition of different H3 variants influences the functional organization of chromatin. Taken together, these findings suggest that dynamic changes in the deposition of H3 variants are critical for chromatin reorganization during epigenetic reprogramming.

## Author Summary

Histones play essential roles in the regulation of chromatin structure and gene expression. The functions of histone H3 non-centromeric variants (H3.1, H3.2, and H3.3) have not yet been fully understood. Little is known about the mammalian-specific H3.1 in terms of deposition pattern and role. We show here that the nuclear deposition of these three variants is dynamically changed after fertilization and in the process of embryo preimplantation development, during which genome reprogramming occurs. H3.3, an active gene marker, is removed from the female pronucleus soon after fertilization, suggesting that the epigenetic marks carried by H3.3 in differentiated oocytes are erased. This process might participate in generating totipotency in early embryos. During the late preimplantation stage, H3.1 appears to prevent H3.3 deposition in inactive chromatin domains. The interplay among deposition of H3 variants likely participates in the functional organization of chromatin. Together, our results suggest that dynamic replacement of histone variants plays important roles in genome remodeling.

## Introduction

Post-translational modifications of core histones contribute to determining chromatin states in transcriptional activation and repression, thereby regulating various cellular functions [1]–[3]. Moreover, in differentiated cells, histone modifications propagate information about genomic function faithfully from one generation of cells to the next [4]–[6].

The discovery of histone variants has led to the emerging notion that alterations in histone modifications and further changes in chromatin structure are induced by exchanges of histone variants [7]–[9]. In mammals, three variants of histone H3, known as H3.1, H3.2, and H3.3, differ primarily in their chromatin deposition patterns and post-translational modifications. Histone H3.1 and H3.2 proteins are expressed during the S phase, and their deposition in chromatin is dependent on DNA replication. In contrast, H3.3 proteins are expressed and deposited in chromatin throughout the cell cycle by DNA replication uncoupled pathways [10], [11]. H3.3 is an evolutionally conserved variant, and its deposition is correlated with transcriptionally active genes [12]-[14]. Accordingly, histone modifications associated with active gene expression are enriched on H3.3 [15]–[17]. H3.3 remains at genetic regions during M phase [10], [18], suggesting that it contributes to the epigenetic inheritance of chromatin states. Moreover, the results of recent studies suggest that the rate of turnover of H3.3 determines the epigenetic state at promoters and regulatory elements [19], [20]. H3.1 is enriched in dimethylated H3 Lys9 (H3K9me2), which is associated with gene silencing and heterochromatin formation [21]. H3.2 is enriched in other histone modifications associated with gene silencing. H3.1 is a mammalian-specific variant and differs from canonical histone H3.2 by only one amino acid (amino acid position 96: Cys/Ser). Although the difference in the deposition pattern or function between these two variants is not yet understood, the difference in expression levels among cell lines and post-translational modifications suggests that H3.1 potentially has different functions from H3.2 [16]. Therefore, the distribution of H3 variants in the genome and their different patterns of modification seem to determine cellular states of differentiation.

Recently, the dynamics of histone variants during fertilization and development has been studied in animals and plants [22]–[25]. H3.3 is incorporated in male pronuclei independently of transcription after fertilization in *C. elegans*, flies, and mice [26]–[29] and is involved in the establishment of heterochromatin in the mouse early embryo [30]. In *Arabidopsis*, the dynamics of H3.3 in zygotes is distinct from that in endosperm [31], and H3 variants are actively removed from the zygote chromatin [32]. These findings suggest that H3 variants may play conserved roles in chromatin remodeling during genome reprogramming during early embryonic life [33]. However, little is known about the changes in deposition pattern of H3 variants and their roles in genome reprogramming during fertilization and preimplantation development.

In the present study, we investigated the deposition patterns of three H3 variants (H3.1, H3.2, H3.3) during oogenesis and preimplantation development in mice. The results showed that H3.3, a marker of active genes, is transiently removed from the maternal genome after fertilization. We also showed that H3 variants differed in the timing of incorporation into genomes and in their nuclear distribution during preimplantation development. The deposition of H3 variants was interactive, and the proper distribution of these variants was found to be important for the pattern of histone modifications, chromatin organization, and preimplantation development.

## Results

### Deposition of H3 variants in DNA replication-dependent or -independent pathways in somatic cells

The amino acid sequences of histones H3.1, H3.2, and H3.3 are quite similar [7], and no H3 variant-specific antibodies are currently available. We therefore constructed vectors expressing Flag epitope-tagged versions of these three variants. First, we confirmed that the Flag-H3.1/H3.2 and Flag-H3.3 show nucleosome assembly only during the S phase and in a cell-cycle-independent manner, respectively. The expression vector encoding each tagged variant was transiently transfected in mouse NIH 3T3 cells (Figure S1). Immunofluorescence staining at 48 h post-transfection showed the transfection efficiency of Flag-H3.1 (17%), Flag-H3.2 (19%), and Flag-H3.3 (27%). Treatment with the DNA synthesis inhibitor aphidicolin from 12 to 48 h post-transfection severely impaired incorporation of Flag-H3.1 (3%) and Flag-H3.2 (5%) into the nuclei. However, this treatment induced only a slight decrease in Flag-H3.3 incorporation into chromatin (22%). These results indicate that the Flag epitope tag did not alter H3 variants' typical modes of incorporation [10], [11].

### Dynamics of H3.3 incorporation in oocytes and embryos before and after fertilization

**Figure 1:**
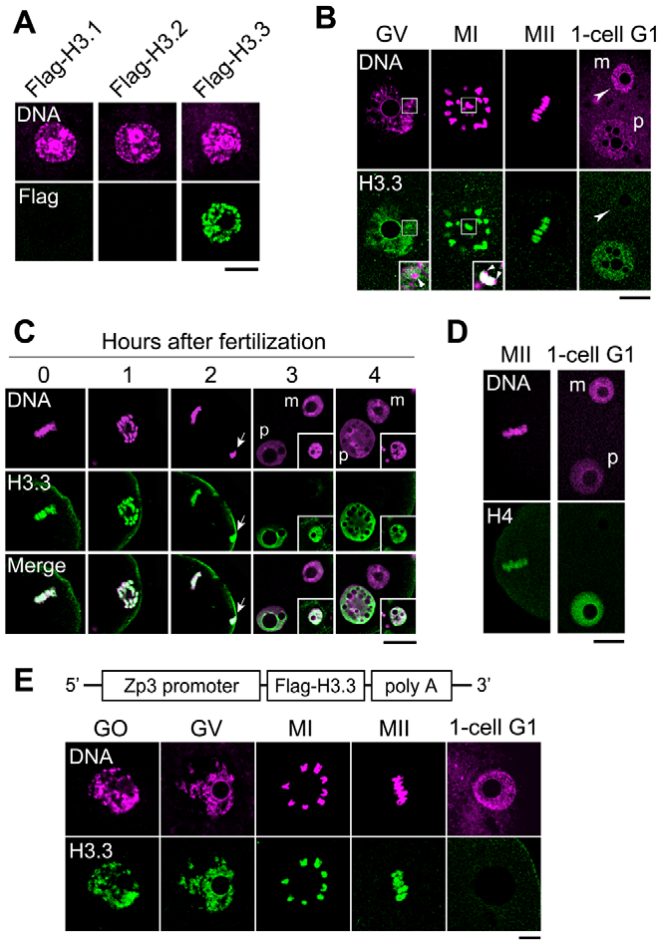
H3.3 disappears from the oocyte genome immediately after fertilization. (A) Deposition of Flag-H3 variants in growing oocytes. Each Flag-H3 variant mRNA was microinjected into the cytoplasm of growing oocytes, and the oocytes were immunostained 24 h later with anti-Flag antibody. DNA was counterstained with PI. Scale bar, 20 µm. (B) Analysis of Flag-H3.3 dynamics before and after fertilization. Fully grown oocytes (GV) were microinjected with Flag-H3.3 mRNA in the presence of IBMX, which inhibits meiotic maturation. Five hours later, Flag-H3.3 was detected in GV-stage oocytes, and 2 h and 18 h after the removal of IBMX it was still present in MI- and MII-stage oocytes, respectively. Merged images are shown in the inset, where the arrows show little or no signal for Flag-H3.3 in the heterochromatic regions. Four hours after fertilization *in vitro* (1-cell G1), Flag-H3.3 was not detected in the whole maternal pronucleus (m, arrowheads), whereas it was detected in the paternal pronucleus (p). Scale bar, 20 µm. (C) Analysis of H3.3 dynamics in the maternal genome during the early one-cell stage. The embryos were collected at 0, 1, 2, 3, and 4 h after insemination and examined for Flag-H3.3 incorporation into the maternal genome. The polar body genome from each embryo is shown by arrows (2 h) or in the inset (3 and 4 h). Scale bar, 20 µm. (D) Analysis of Flag-H4 dynamics before and after fertilization. The nuclear deposition of Flag-H4 was examined using the same procedure as for Flag-H3.3 in (B). Flag-H4 was not detected in the maternal pronucleus (m, arrowheads), but was detected in the paternal pronucleus. (E) The nuclei of growing oocytes (GO) and GV-stage oocytes, the chromosomes of MI- and MII-stage oocytes, and the female pronuclei of one-cell G1 embryos (1-cell G1) from a Zp3-Flag-H3.3 transgenic mouse were analyzed by immunostaining with anti-Flag antibody. Scale bar, 10 µm.

To examine the dynamics of H3 variants during oogenesis, we injected mRNAs encoding the Flag-H3 variants into the cytoplasm of oocytes. In growing oocytes from 12-day-old female mice, which do not experience DNA synthesis, we could not detect Flag-H3.1 and Flag-H3.2 deposition in chromatin (Figure 1A). In contrast, an intense Flag-H3.3 signal was observed throughout the nucleus (Figure 1A), indicating that incorporation of H3.3 into chromatin takes place during oogenesis. In fully grown oocytes and in the chromosomes during meiosis I, an intense Flag-H3.3 signal was detected, primarily in euchromatic regions (identified by weak DNA staining) (Figure 1B), but little or no signal was detected in the heterochromatic pericentromeres (Figure 1B). These results suggest that H3.3 is deposited in transcriptionally active loci and that the epigenetic information it carries is maintained during meiosis.

To address whether H3.3 is inherited from the mature oocyte by the zygote, Flag-H3.3 mRNA was injected into the cytoplasm of fully grown oocytes. We observed the H3.3 dynamics after fertilization of mature oocytes. Maternally provided Flag-H3.3 was loaded into the paternal pronucleus (Figure 1B), as observed previously [28], [29], [34]. In contrast, the signal from maternally expressed Flag-H3.3 decreased markedly in the female pronucleus (Figure 1B). A precise observation at hourly intervals revealed that Flag-H3.3 was no longer detected around the time of maternal pronucleus formation, while it remained in polar bodies (Figure 1C). The loss of H3.3 was also observed in parthenogenetically activated oocytes (Figure S2), suggesting that the paternal genome has no influence on the disappearance of H3.3 from the maternal genome after fertilization. Finally, since histone H4 is a binding partner of H3 in nucleosomes, we examined the dynamics of H4 after fertilization by microinjecting Flag-H4 mRNA into oocytes. Our results showed that H4 that had been incorporated into the oocyte genome was not present after fertilization (Figure 1D), indicating that H3.3 is removed in the form of H3.3/H4 dimers or tetramers.

To examine whether H3.3 depletion is a post-fertilization-specific event that is not cell-cycle dependent after the M phase, we examined Flag-H3.3 deposition after the M phase in the next cell cycle, *i.e.*, the transition from the one-cell M phase to the two-cell G1 phase. We found that Flag-H3.3 deposited in the mitotic chromosomes of one-cell embryos was maintained in the G1-phase nuclei of two-cell embryos (Figure S3). These results indicate that loss of H3.3 from the zygotic genome occurs specifically after the completion of the second meiosis.

We observed similar dynamics in a transgenic mouse expressing Flag-H3.3 from the *Zp3* promoter specifically during oocyte growth (Figure 1E). These results strongly suggest that fertilization triggers the removal of maternal H3.3 accumulated in the female pronucleus. We hypothesize that erasure of the epigenetic information carried by maternal H3.3 participates to initiate the new pattern of gene expression in the totipotent zygote.

**Figure 2:**
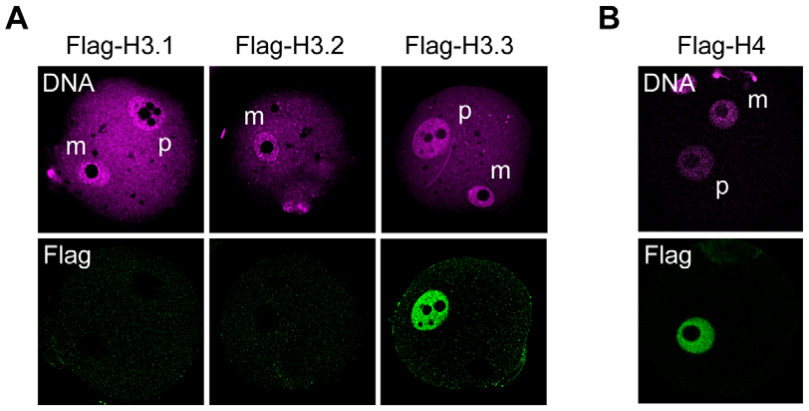
Incorporation of H3 variants and H4 into nuclei at an early stage after fertilization. MII-stage oocytes were microinjected with mRNA encoding a Flag-H3 variant or Flag-H4, and then fertilized 2 h later. Four hours after fertilization, one-cell embryos were stained with anti-Flag antibodies; DNA was stained with DAPI. (A) No signal for any Flag-H3 variant was detected in the maternal pronuclei of early one-cell embryos. (B) Flag-H4 was also undetectable in the maternal pronucleus, although it was clearly detected in the paternal pronucleus. m, maternal pronucleus; p, paternal pronucleus.

Finally, we sought to determine which H3 variants replace the H3.3 removed from the maternal genome after fertilization. We microinjected mRNA encoding either of the Flag-H3 variants and followed the incorporation of these proteins into the maternal genome after fertilization. Four hours after fertilization, no incorporation of Flag-H3.1 or -H3.2 was observed (Figure 2A). Therefore, it is possible that another unknown H3 variant is specifically incorporated soon after fertilization. To address this hypothesis, we examined the incorporation of histone H4 on the basis that if an H3 variant is incorporated, H4 should accompany it. Metaphase II (MII)-stage oocytes were microinjected with mRNA encoding Flag-H4 and then examined for H4 incorporation into the maternal genome 4 h after fertilization. We did not detect Flag-H4 incorporation into the maternal genome soon after fertilization, although its incorporation into the paternal genome was clearly detected (Figure 2B). These results suggest that no replacement occurs when H3.3 is removed, and that genomic regions from which H3.3 has been removed remain nucleosome-free for a while after fertilization.

### Changes in Flag-H3 variants' incorporation during preimplantation development

**Figure 3:**
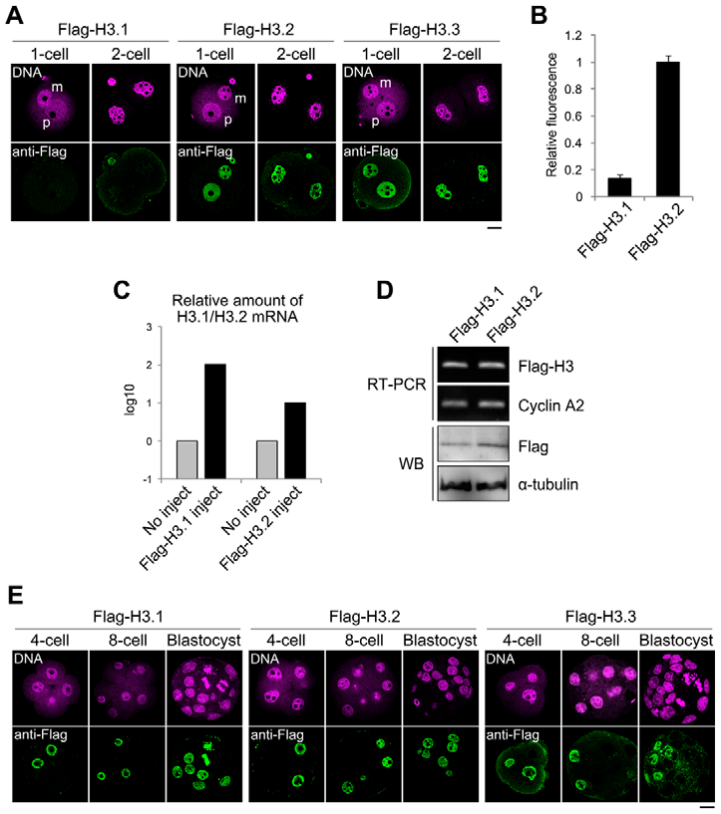
Incorporation of Flag-H3 variants into nuclei in preimplantation embryos. (A) Immunofluorescence analysis of Flag-H3 variants in one- and two-cell embryos. MII-stage oocytes were microinjected with each Flag-H3 variant mRNA and then fertilized 2 h later. Staining with anti-Flag antibody was observed in the one- and two-cell embryos at 12 h and 28 h post-fertilization, respectively. m, maternal genome; p, paternal genome. The DNA was stained with PI. Scale bar, 20 µm. (B) The fluorescence intensity of Flag-H3.1 and Flag-H3.2 in the two-cell embryo was determined as described in the Materials and Methods section. The numbers of nuclei examined were 80 and 64 for the Flag-H3.1- and Flag-H3.2-injected embryos, respectively. (C) Quantitative RT-PCR analysis for H3.1 or H3.2 mRNA in two-cell embryos injected with or without Flag-H3.1 or Flag-H3.2 mRNA prior to fertilization. RT-PCR was performed using the primer pairs for the H3.1 or H3.2 open reading frames, which measured the total amounts of endogenous and injected mRNAs. The target mRNA level was normalized to the amount of exogenous rabbit α-globin mRNA in the same sample. The average value of duplicate experiments is shown. (D) Analysis of Flag-H3.1 and Flag-H3.2 mRNA and protein level in two-cell embryos by RT-PCR and immunoblotting (WB). RT-PCR was performed using a common primer pair recognizing exogenous Flag-H3.1 and Flag-H3.2 mRNA, but not endogenous H3.1 or H3.2. Endogenous cyclin A2 mRNA served as a control. Flag-H3.1 and Flag-H3.2 proteins were detected by immunoblotting with anti-Flag antibody. The relative values of the band densities of H3.1 vs. H3.2 were 0.63 vs. 1.0. Antibody against α-tubulin was used for the loading control. The experiments were conducted twice with similar results. (E) Immunofluorescence of Flag-H3 variants in late preimplantation embryos. Each Flag-H3 variant mRNA was microinjected into one blastomere of two-cell embryos. Therefore, signals for the Flag-H3 variants were detectable in half of the blastomeres. Deposition of each H3 variant in embryos at the four-cell, eight-cell, and blastocyst stages was examined at 44 h, 56 h, and 80 h post-fertilization, respectively. Scale bar, 20 µm.

Next, we examined the temporal and spatial dynamics of the H3 variants during preimplantation development. We injected mRNAs encoding the Flag-H3 variants into the cytoplasm of MII-stage oocytes prior to fertilization. The oocytes were fertilized *in vitro* and then cultured until the embryos had undergone DNA replication and had reached the late one-cell stage (12 h) or late two-cell stage (28 h). Flag-H3.2 and Flag-H3.3 accumulated in both pronuclei in one-cell embryos and in the nuclei of two-cell embryos (Figure 3A). Surprisingly, the signal for Flag-H3.1 was significantly weaker during this period (Figure 3A, 3B). These data suggested that incorporation of H3.1 was less efficient than that of H3.2 in early embryos or that a specific degradation mechanism targeted the Flag-H3.1 mRNAs or protein. Quantitative RT-PCR showed that the exogenous histone mRNA injected before fertilization still remained in large amounts compared with the endogenous histone mRNA at the two-cell stage (Figure 3C). We next examined the Flag-H3.1 protein level by immunoblotting. The amounts of Flag-H3.1 protein was smaller than that of H3.2 but their difference was not prominent (37% smaller; Figure 3D), indicating that Flag-H3.1 was translated at the early stages, but its incorporation into chromatin was limited. The reason why the amount of Flag-H3.1 protein was slightly smaller is likely because histone proteins that are not packaged into chromatin are efficiently degraded [35]. We confirmed these limitation of H3.1 incorporation by using Flag-tagged histone mRNA that was synthesized from a different construct and efficiently translated in mouse oocytes and embryos (Figure S4A, S4B) [36]. Moreover, using mRNA encoding EGFP-tagged H3.1 and H3.2, we detected a strong signal for EGFP-H3.2 in the chromatin of one-cell embryos. In contrast, the EGFP-H3.1 signal was weak (Figure S4C). Together, we conclude that much less H3.1 than H3.2 is incorporated into early-stage embryos.

We examined the cycle-dependent incorporation of H3 variants during the one-cell stage (Figure S5). The results showed that Flag-H3.2 was incorporated into the nucleus only during the S phase. This incorporation was strictly DNA replication dependent, as aphidicolin treatment inhibited the incorporation. Flag-H3.3 was incorporated not only during the S phase but also during the G2 phase. Aphidicolin treatment inhibited incorporation only during the S phase but not during the G2 phase, indicating that H3.3 was incorporated by both DNA replication-dependent and -independent mechanisms.

To determine whether the limitation on H3.1 incorporation persists in late preimplantation embryos until the blastocyst stage, we injected the Flag-H3 variant mRNAs into the cytoplasm of blastomeres of late two-cell embryos. All Flag-H3 variants, including Flag-H3.1, strongly labeled the four-cell embryo nuclei, and the signals were also detected in the morula- and blastocyst-stage embryos (Figure 3E). In conclusion, our results suggest that H3.2 and H3.3 accumulate in the nuclei of embryos throughout the preimplantation stage, *i.e.*, from the one-cell stage through the blastocyst stage, whereas H3.1 is incorporated less efficiently into nuclei until the two-cell stage.

### Localization of Flag-H3 variants at the early and late preimplantation stages

H3.1 is suggested to be associated with heterochromatin formation, whereas H3.3 is preferentially localized in euchromatin [7]. It was thus possible that the absence of H3.1 incorporation was correlated with a peculiar organization of the chromatin during the first two cell cycles after fertilization. Nuclear regions are classified into euchromatin and heterochromatin according to their DNA staining patterns, with regions of densely stained DNA defined as heterochromatic sites [37]-[39]. A dynamic reorganization of chromatin occurs during preimplantation development (Figure S6). In one- or two-cell embryos, the chromatin is decondensed, and the heterochromatin domains are mostly confined to the peripheries of nucleoli. As the embryos develop into the blastocyst stage, several well defined heterochromatin foci become detectable.

**Figure 4:**
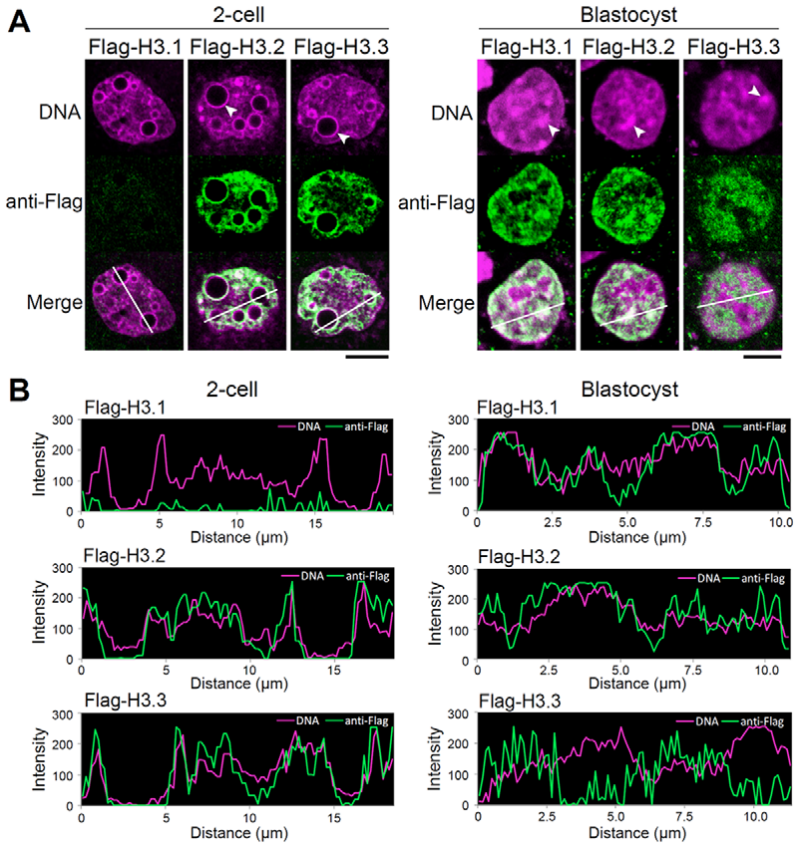
Intranuclear distribution of H3 variants changes dynamically during preimplantation development. (A) Immunodetection of Flag-H3 variants at the two-cell and blastocyst stages. Heterochromatin domains, which are characterized by DNA-dense foci, are confined to the peripheries of nucleoli at the two-cell stage, whereas they localize at discrete foci in the nucleoplasm at the blastocyst stage. Arrowheads indicate the typical heterochromatin foci. Scale bar, 10 µm (two-cell stage), 5 µm (Blastocyst). (B) Fluorescence intensity profiles of Flag-H3 variants and DNA in the nucleus of two-cell and blastocyst-stage embryos. Lines were drawn on the images of nuclei (see merged images in (A)), and the pixel intensities for Flag-H3 variant staining (green) and DNA staining (magenta) were quantified along the lines. The horizontal axis represents the distance from the starting (farthest left) point of the analysis on the line.

To define the sites of variant deposition during early and late preimplantation development, we compared the nuclear localization of Flag-H3 variants in the two-cell vs. blastocyst stages (Figure 4A). In two-cell embryos, Flag-H3.1 did not accumulate in any chromatin regions, whereas in blastocysts, intense Flag-H3.1 signals were detected in both euchromatic and heterochromatic foci. Localization of Flag-H3.2 was observed in euchromatin and heterochromatin at both the two-cell and blastocyst stages. At the two-cell stage, we also detected Flag-H3.3 labeling throughout the entire chromatin region, including the perinucleolar heterochromatin, but at the blastocyst stage, it was almost entirely deposited in euchromatic regions, with little or no deposition in heterochromatic regions. We confirmed these co-localization patterns of Flag-histone variants with heterochromatic regions using fluorescence in situ hybridization (FISH) for major satellites that represent the predominant heterochromatic regions (Figure S7). Additionally, we found that all of the variants were uniformly distributed in both heterochromatin and euchromatin regions at the four-cell stage (data not shown), suggesting that the four-cell stage may be the transition period for chromatin reorganization. These results suggest that H3.3 localization in chromatin changes dynamically during preimplantation development. We examined the localization of these variants using fluorescence intensity profiling (Figure 4B). As the DNA signal intensity increased, that of Flag-H3.1 in blastocysts and Flag-H3.2 in two-cell embryos and blastocysts also tended to increase. Although the intensity of the Flag-H3.3 signal was also positively correlated with that of DNA in two-cell embryos, a negative correlation for the Flag-H3.3 signal was observed in the DNA-dense heterochromatic regions in the blastocysts.

We concluded that in early preimplantation embryos H3.3 localized throughout the chromatin, including in the heterochromatic domains where H3.1 would be expected. Only in late preimplantation embryos did H3.1 and H3.3 assume the typical distribution in euchromatin and heterochromatin, respectively. These observations suggested that early preimplantation embryos experienced a mechanism either limiting H3.1 incorporation or promoting H3.3 incorporation in heterochromatic regions.

### CAF-1 p150 knockdown induces alteration of H3 variants' distribution and impaired heterochromatin formation during late preimplantation development

**Figure 5:**
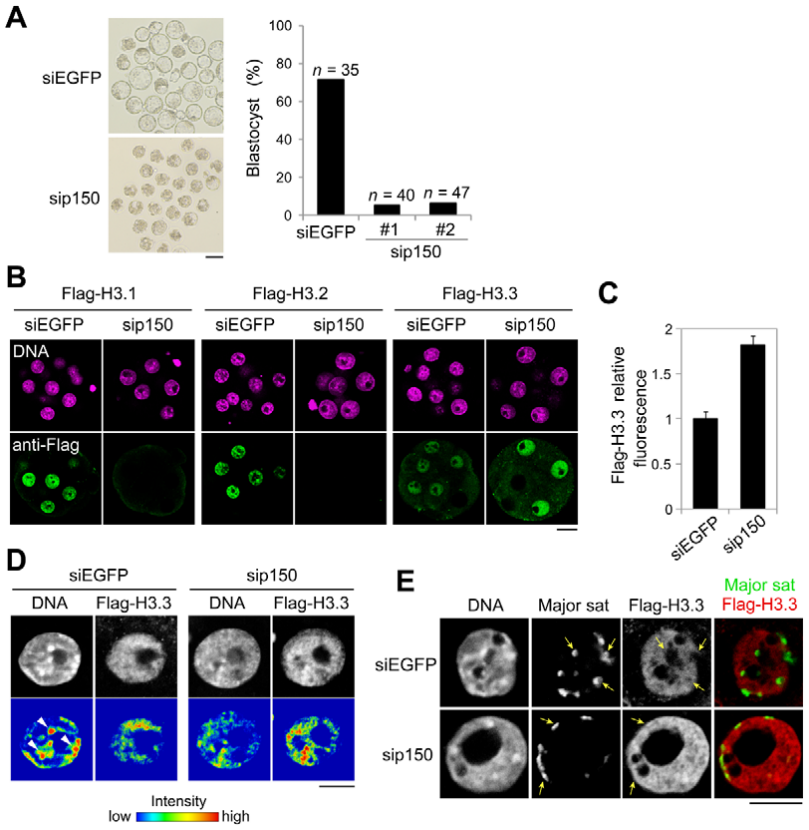
Knockdown of CAF-1 p150 impairs distribution of H3 variants and leads to the inhibition of heterochromatin formation. One-cell embryos were microinjected with siRNA targeting CAF-1 p150 (sip150#1 or sip150#2) or with siRNA targeting EGFP (siEGFP) as a control. (A) The morphology of embryos treated with siRNA at 96 h post-fertilization and the percentage of treated embryos that successfully progressed to the blastocyst stage are shown; *n* indicates the number of embryos examined. Scale bar, 100 µm. (B) The siRNA-treated embryos were microinjected with each Flag-H3 variant mRNA at the two-cell stage. The mRNAs were microinjected into one blastomere of two-cell embryos. Signals for the Flag-H3 variants were detectable in half of the blastomeres. Embryos that developed to the morula stage were immunostained with anti-Flag antibody, and the DNA was stained with DAPI. Scale bar, 20 µm. (C) Quantitative analysis of Flag-H3.3 staining intensity in the siRNA-treated morulae. The numbers of nuclei examined were 69 and 50 for the siEGFP- and sip150-treated embryos, respectively. (D) Fluorescence intensity analysis of DNA and Flag-H3.3 in siRNA-treated morulae. Arrowheads indicate the typical chromatin regions in which DNA is densely stained. Scale bar, 10 µm. (E) Immuno-DNA-FISH analysis for localizing major satellites and Flag-H3.3 in the nucleus of sip150-treated embryos. Arrows indicate the typical major satellites. Scale bar, 10 µm.

The dynamic change in H3.1 deposition in chromatin between early and late preimplantation suggested that proper deposition of H3.1 variants was important for heterochromatin formation. To address this hypothesis, we designed the experiments to inhibit H3.1 incorporation during preimplantation development by limiting the expression of the H3.1 chaperone subunit CAF-1 p150 (sip150) [11]. CAF-1 p150 knockdown significantly affected preimplantation development. Although approximately 70% of the control embryos injected with siRNA against EGFP (siEGFP) developed into blastocysts, only ∼10% of the sip150-injected embryos reached the blastocyst stage (Figure 5A). In most of the latter group of embryos, development stopped at the eight- to 16-cell stage, *i.e.*, in the early morula stage. In these embryos, the relative amount of DAPI-dense regions was reduced (Figure S8, *t*-test; *P*<0.005), and nucleus size was increased (1.4-fold compared with the control, *t*-test; *P*<0.001). These phenotypes are consistent with those in CAF-1 p150-knockout mouse embryos [40]. In these embryos, heterochromatin formation was impaired, and the nuclear deposition of the heterochromatin-related protein HP1alpha was reduced.

In sip150-injected embryos, we found that the incorporation of not only Flag-H3.1 but also of Flag-H3.2 was completely lost (Figure 5B), suggesting that CAF-1 is also an H3.2 chaperone. In contrast, sip150 treatment resulted in a significant increase in Flag-H3.3 incorporation (Figure 5B, 5C). In addition, Flag-H3.3 tended to be deposited in the relatively DNA-dense regions in those nuclei, whereas it was preferentially deposited in DNA-sparse regions in control nuclei (Figure 5D). Although in the control embryos, the major satellites (detected by immuno-FISH) were localized in the nuclear interior region and not co-localized with Flag-H3.3, in sip150-injected embryos, the major satellites were localized at the nuclear periphery overlapping with a uniform distribution of Flag-H3.3 (Figure 5E). These results suggest that H3.1 and H3.2 incorporation by p150 CAF-1 competes with H3.3 deposition and indirectly regulates heterochromatin distribution during preimplantation development.

### Incorporation of H3 variants affects the pattern of histone modifications in preimplantation embryos

**Figure 6:**
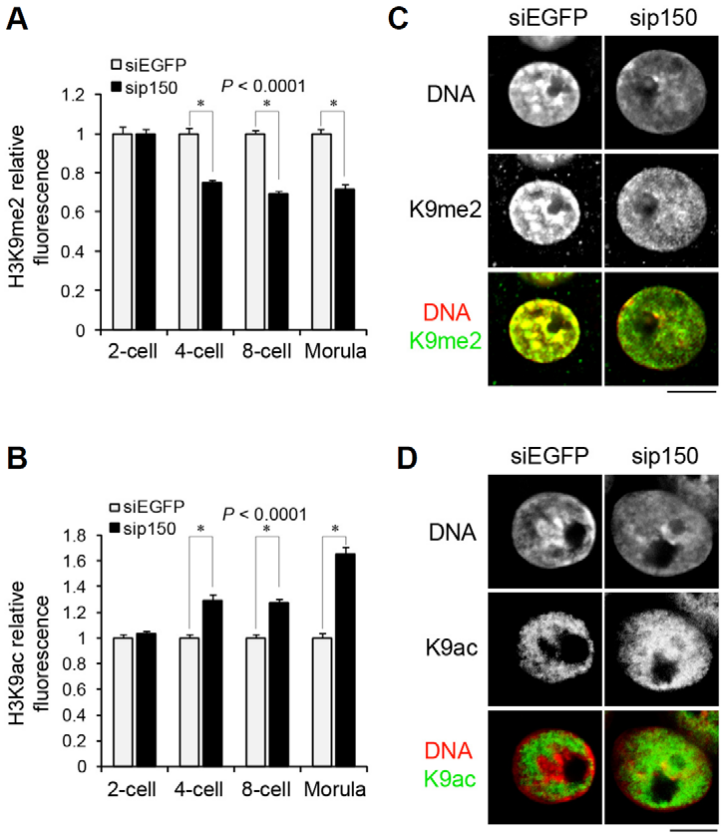
Distribution of H3 variants affects the pattern of histone modifications at late preimplantation stages. (A, B) Quantitative analysis of the fluorescence intensity of H3K9me2 (A) and H3K9ac (B) in entire nuclei of sip150-treated embryos. Embryos that developed to the two-, four-, eight-cell, and morula stage were immunostained with anti-H3K9me2 and anti-H3K9ac antibodies, and the fluorescence intensities were determined as described in the Materials and Methods section. More than 45 nuclei were examined for each group. (C, D) Immunostaining analysis for H3K9me2 (C) and H3K9ac (D) in the nuclei of siEGFP- and sip150-treated morulae. Representative images of the embryos are shown. Scale bar, 10 µm.

We examined the relationship between replacing H3 variants and changes in H3 post-translational modifications during preimplantation development. The H3K9me2 level has been reported to decrease passively during DNA replication at the one- and two-cell stages and to increase from the four-cell stage [41]. Because H3.1 is enriched in H3K9me2 [16], and H3.1 incorporation only begins at the four-cell stage, as shown above, it is possible that the observed increase in H3K9me2 was caused by the incorporation of H3.1 from the four-cell stage. To address this issue, we investigated H3K9me2 levels in sip150-treated embryos. The H3K9me2 level in the four-cell through morula stages was significantly lower in sip150-treated embryos than in control embryos (Figure 6A), suggesting that H3.1 incorporation led to an increase in H3K9me2 in late preimplantation embryos. In contrast, the level of acetylated H3K9 (H3K9ac), which is enriched on H3.3, was higher in sip150-treated embryos than in control embryos (Figure 6B). This difference seemed to correlate with an increase in H3.3 incorporation into sip150-treated embryos (Figure 5C). Close observation of the nuclei in the embryos at the morula stage revealed that H3K9me2 was uniformly distributed in the nucleus in sip150-treated embryos, whereas it was mostly concentrated in heterochromatic regions in siEGFP-treated control embryos (Figure 6C). H3K9ac was also uniformly distributed in the nucleus except for the nuclear peripheral region in sip150-treated embryos, but it was not localized in heterochromatic regions in the control embryos (Figure 6D). Therefore, disequilibrium in H3 variants by p150 knockdown may lead to changes in the level and distribution of histone modifications in the nucleus. These results suggest that genome-wide replacement of H3 variants contributes to the remodeling of epigenetic modifications during preimplantation development.

## Discussion

To the best of our knowledge, this study is the first to investigate the nuclear deposition of all three histone H3 variants (H3.1, H3.2, H3.3) in mammals. We observed both spatial and temporal differences in the patterns of incorporation of each histone variant into the genome after fertilization. We also found evidence that the deposition of histone H3 variants in chromatin is interactive and that proper distribution of these variants is crucial for chromatin reorganization during preimplantation development.

The differentiated somatic cells express the same subsets of genes as did parent cells after cell divisions. It was proposed that H3 variants participate in the maintenance of epigenetic information [10], [18]. We assumed that the reprogramming of gene expression probably requires dynamic changes in H3.3 distribution in the maternal genome after fertilization. In the present study, we showed that a transient loss of H3.3 nucleosomes occurs specifically at the G1 phase of the zygote. These findings suggest that the epigenetic information encoded by H3.3 in growing oocytes is erased after fertilization. Therefore, genome reprogramming may involve erasing cell memory by removing H3.3 to remodel H3.3 distribution in the maternal genome to generate totipotent embryos.

Recently, Ingouff et al. [32] showed that a loss of H3.3 occurs after fertilization in *Arabidopsis*, suggesting that this event is evolutionarily conserved. They also concluded that that the incorporation of zygotic histones accompanies the removal of H3.3. However, our results showed that although histone H4, a binding partner of all H3 variants, was incorporated into the paternal genome, it was not incorporated into the maternal genome before the first S phase. Therefore, we suggest that genomic regions from which H3.3 has been removed remain transiently free of nucleosomes after fertilization.

H3.3 is incorporated into the euchromatic regions of fully grown oocytes (Figure 1A), although no transcription occurs in these oocytes [42]. This result is consistent with the finding that H3.3 replacement occurs continuously at regulatory regions, regardless of whether the gene is active or not [43]. It is thought that the replication-independent incorporation of H3.3 is a marker of nucleosome turnover, and that rapid turnover at sites involved in gene regulation is likely to preserve epigenetic information [20]. After fertilization, H3.3 which had been incorporated into chromatin during oocyte growth was globally removed from the genome and no incorporation of H3 variants was detected before the first S phase, suggesting that nucleosome turnover does not occur during this period.

As embryos develop into the blastocyst stage, chromatin is reorganized into well defined heterochromatin foci. Our results suggest that changes in the deposition of H3.1 and H3.3 are involved in heterochromatin reorganization during preimplantation development. H3.3 was localized to pericentromeric heterochromatin in the periphery of nucleoli at an early stage. Early-stage embryos strongly express transcripts from major satellites on pericentromeres that play critical roles in the formation of heterochromatin and embryo development [44]. A recent study showed that methylation of K27 on H3.3 is involved in the formation of heterochromatin and pericentromeric transcription in early embryos [30]. Therefore, heterochromatin containing H3.3 seems to regulate transcription in pericentromeric regions at an early stage. Moreover, limited incorporation of H3.1 may allow the increased localization of H3.3 to genomic sites, thereby enabling highly decondensed open chromatin to be formed in early-stage embryos. H3.1 incorporation from the four-cell stage seems to be responsible for the formation or maintenance of heterochromatin at the late stages, while H3.3 is simultaneously deposited into the regions other than heterochromatin to form the euchromatin region. Importantly, these H3 variants interact with one another, as the inhibition of H3.1 and H3.2 incorporation caused the increased incorporation of H3.3 and its abnormal distribution pattern at the late preimplantation stage.

The localization of H3.3 at pericentromeric heterochromatin in early preimplantation embryos may be regulated by specific chaperones. Recent studies showed that ATRX and DAXX are involved in the HIRA-independent localization of H3.3 at heterochromatic regions such as the pericentromere and telomere [13], [45], [46]. Importantly, in the absence of DAXX, CAF-1 can recruit H3.3 into chromatin, but the deposition patterns were changed [46], suggesting that the balance of activities in various histone chaperones affects the deposition patterns of histone variants. Therefore, the change in chaperone activity may regulate the change in histone variants' deposition into pericentromeric heterochromatin during preimplantation development. DNA hypomethylation during the early preimplantation stage [47] may also be related to H3.3 deposition in heterochromatin, as the decrease in DNA methylation induces the accumulation of H3.3 in pericentromeric heterochromatin [48]. Alternatively, H3.3 may only be incorporated into pericentromeric regions in the paternal genome when protamines are exchanged with histones soon after fertilization because other non-centromeric H3 variants cannot be incorporated into the genome before the first S phase in zygotes.

We have shown that the patterns of nuclear deposition are different between H3.1 and H3.2 in early preimplantation embryos: H3.1 is less efficiently incorporated than H3.2. This suggests that the functions of these two variants differ. H3.1, but not H3.2, may be involved in the limitation of chromatin plasticity or totipotency during preimplantation development. Supporting these results, Hake et al. showed that compared with other variants, H3.1 is more abundant in adult tissue cells and less abundant in embryonic-derived cells in humans [16]. They also suggested that H3.1 cysteine 96 potentially forms intermolecular disulfide bonds in different nucleosomes, leading to chromatin condensation and heterochromatin formation [7]. Therefore, mammalian-specific variant H3.1 may have a function to generate a more complicated chromatin state during mammalian development.

Inconsistent with our results, Santenard et al. [30] recently showed that H3.1 was incorporated into whole nuclei at the same level as H3.3 in one- and two-cell embryos using C-terminally EGFP-tagged histone mRNA. We obtained similar results when C-terminally Flag- or EGFP-tagged versions were used (data not shown). Thus, the discrepancy in the nuclear deposition of H3.1 seems to have stemmed from the difference in the position where the tag was fused. However, we found using ES cells that N-terminally Flag-tagged histones formed proper nucleosome [49] and were modified in the similar manner to C-terminally tagged histones [50] (Figure S9), suggesting that there is an early embryo-specific mechanism regulating the chromatin incorporation of H3.1.

The mechanism for the limitation of H3.1 incorporation into the nucleus at the one- and two-cell stages remains unclear. Although no specific H3.2 chaperone has been identified, a recent study found that H3.1 and H3.2 differed in their post-translational modification patterns [16], suggesting the existence of specific H3.1 or H3.2 chaperones that selectively incorporate H3.1 or H3.2 into different genomic regions. Therefore, the limitation of H3.1 incorporation during the one-cell and two-cell stages may be regulated by these chaperones.

Our results also suggest that histone replacement causes changes in histone post-translational modifications during preimplantation development. Supporting this hypothesis is the finding that the decrease in H3.1 deposition and the reciprocal increase in H3.3 by CAF-1 knockdown resulted in hypomethylation and hyperacetylation of H3K9 from the four-cell stage. Furthermore, we previously showed that dimethylation of H3 lysine-79 (H3K79), a marker of gene activity that is preferential to H3.3, is lost from the maternal genome soon after fertilization [51], possibly due to the removal of H3K79-dimethylated H3.3 after fertilization. Although the mechanism by which the modification patterns differ between H3.1 and H3.3 in chromatin is unknown, differences in the modification status of non-nucleosomal H3.1 versus H3.3 may influence the final patterns of their modifications within chromatin [50].

Taken together, these findings lead us to propose that when cells greatly alter their gene expression patterns, such as in genome reprogramming during gametogenesis and preimplantation development, drastic changes in many varieties of histone modification occur, possibly through histone replacement rather than changes in the activities of histone-modification enzymes. To alter the pattern of numerous histone modifications during the process of reprogramming, marked changes in the activities of a wide variety of these enzymes would be required. On the other hand, the replacement of only a few histone variants would enable global histone modifications. Consistent with this hypothesis, two recent reports revealed chromosome-wide or genome-wide replacement of H3 variants in the XY body and in primordial germ cells, in which patterns of gene expression and histone modifications also change dynamically [52], [53]. Therefore, profiling the composition of nucleosomal histone variants in the genome, which could directly determine the pattern of histone modifications, is essential for understanding the molecular basis of cellular identity.

## Materials and Methods

### Ethics statement

All procedures using animals were reviewed and approved by the University of Tokyo Institutional Animal Care and Use Committee.

### Mice

Three-week-old female BDF1 (C57BL/6 × DBA2) mice were purchased from CLEA Japan. Adult male and female C57BL/6 and ICR mice and male DBA2 mice were purchased from Japan SLC. Twelve-day-old female BDF1 mice were prepared by mating female C57BL/6 mice and male DBA2 mice in our laboratory.

### Oocyte collection and culture

Growing oocytes were collected from the ovaries of 12-day-old female BDF1 mice and cultured in α-minimum essential medium (Gibco) supplemented with 5% fetal bovine serum (Invitrogen) in a humidified 5% CO_2_ atmosphere at 38°C.

Fully grown oocytes containing a germinal vesicle (GV) were collected from 3-week-old female BDF1 mice. The ovaries were removed from the mice 47 h after injection of 5 U of pregnant mare's serum gonadotrophin (PMSG; Sankyo). GV-stage oocytes were collected and incubated in Waymouth's medium [54] containing 0.2 mM 3-isobutyl-methylxanthine (IBMX) in a humidified 5% CO_2_ atmosphere at 38°C. After transfer into IBMX-free medium, the oocytes spontaneously resumed and completed meiosis I and thereafter were arrested at the MII stage.

MII-stage oocytes were collected from 3-week-old female BDF1 mice after superovulation induced by consecutive injections of 5 U of PMSG followed by 5 U of human chorionic gonadotropin (hCG; Sankyo) 46–48 h later. Cumulus–oocyte complexes were collected from ampullae of oviducts 14–16 h after hCG injection.

### In vitro fertilization and embryo culture

MII stage oocytes were fertilized in vitro as previously described [55] and cultured in potassium simplex optimized medium (KSOM) [56] containing 3 mg/ml BSA. Some embryos were transferred to KSOM containing 3 µg/ml aphidicolin (Sigma–Aldrich) 5 h after insemination to inhibit DNA synthesis at the one-cell stage.

### In vitro synthesis and microinjection of mRNA

The coding regions encoding histones H3.1 (Hist1h3g) and H3.2 (Hist1h3f) were amplified by PCR from the total cDNA of GV-stage oocytes. The construct containing the histone H3.3 coding region was a gift from K. Ahmad [10]. A 5′ Flag epitope tag was added to the histone cDNAs by PCR using a primer encoding the eight-amino-acid Flag epitope with a linker polypeptide (GGSGG). To exclude the regulation of untranslated regions, only open reading frames of Flag-H3 variants were cloned into vector pCRII using a TOPO TA cloning kit (Invitrogen), and all constructs were verified by DNA sequence analysis. After the fusion constructs were linearized, the capped mRNAs were made by *in vitro* transcription using an SP6 or T7 mMASSAGE mMACHINE kit (Ambion). Poly(A) tails were added using a Poly(A) Tailing Kit (Ambion). Synthesized mRNAs were purified from enzymatic reactions according to the manufacturer's instructions, diluted in nuclease-free water, and stored at −80°C. Flag-H4, EGFP-H3.1, EGFP-H3.2, and non-tagged histone H3.1 and H3.2 mRNAs were synthesized in the same manner.

Microinjection was performed under an inverted microscope (Eclipse TE300, Nikon) equipped with a micromanipulator (Narishige) and microinjector (IM300, Narishige). Oocytes or embryos were transferred to HEPES-buffered KSOM and injected with ∼10 pl of mRNA (100 ng/μl) into the cytoplasm using narrow glass capillaries (GC100 TF-10; Harvard Apparatus).

To analyze the dynamics of H3 variants from the four-cell to the blastocyst stage, each Flag-H3 variant mRNA was microinjected into one blastomere of two-cell embryos.

### Transgenic mice

The pCAGGS vector [57] (a gift from H. Tojo) was used to generate Zp3-Flag-H3.3 transgenic mice. Flag-H3.3 was subcloned from the pCRII vector into the pCAGGS vector in which the CAG promoter cassette was replaced with the ∼1.5-kb Zp3 promoter obtained from the Zp3 vector [58] (a gift from R. M. Schultz). The Zp3-Flag-H3.3 plasmid was linearized and subjected to electrophoresis. Gel-purified transgenes were injected into the pronuclei of fertilized eggs derived from inbred C57BL/6 mice. Embryos that had reached the two-cell stage were transferred into the oviducts of 8- to 10-week-old ICR female mice mated during the previous night with vasectomized ICR male mice.

### Somatic cell culture and transfection

NIH 3T3 cells were grown in Dulbecco's modified Eagle's medium (Sigma–Aldrich) supplemented with 0.4% penicillin–streptomycin (Gibco) and 10% fetal bovine serum (Invitrogen). For transient transfection, the Flag-H3 variant coding sequences were subcloned from the pCRII vector into the pEGFP-N1 expression vector, deleting the EGFP cassette. Cells were transfected with the expression vector using Transfast reagent (Promega) according to the manufacturer's instructions. In some experiments, the cell medium was supplemented with aphidicolin (10 µg/ml) 12 h after transfection, and 48 h after transfection, the cells were tested for tagged histone expression using immunostaining.

### Immunofluorescence

NIH 3T3 cells, oocytes, and embryos were fixed with 3.7% paraformaldehyde (Wako) in phosphate-buffered saline (PBS) for 30 min (NIH 3T3 cells) or 1 h (oocytes and embryos). After washing with PBS/0.1% BSA, they were permeabilized with 0.5% Triton X-100 (Wako) in PBS for 15 min and then incubated overnight at 4°C with anti-Flag M2 mouse monoclonal antibody (Sigma–Aldrich) or rabbit antibody against H3K9me2 (Upstate) or H3K9ac (Cell Signaling) diluted 1∶100 in PBS/0.1% BSA. The cells were washed and incubated with Alexa-Flour 488- or 568-conjugated secondary antibodies (Molecular Probes) for 2 h at room temperature. DNA was visualized with either 4′,6-diamidino-2-phenylindole (DAPI; Dojindo) or propidium iodide (PI; Sigma–Aldrich).

Confocal digital images were obtained using a laser-scanning microscope (LSM510; Zeiss). Fluorescence intensity profiles were analyzed using Zeiss LSM software. Semi-quantitative analysis of the fluorescence signals was conducted using the NIH Image program, as previously described [59]. Briefly, the pixel value/unit area was measured for the nucleus, and the value for the cytoplasm was subtracted as background. The value obtained was multiplied by the nuclear area to yield the total amount of fluorescence in the nucleus.

Approximately 10 oocytes/embryos were examined in every group, and each experiment was repeated at least three times.

### Immuno–DNA FISH

Embryos expressing Flag-H3 variants were immunostained with anti-Flag rabbit polyclonal antibody (Sigma–Aldrich) followed by Alexa-Flour 568-conjugated secondary antibody (Molecular Probes). FISH analysis was performed based on the procedure described by [60]. The major satellite probe was synthesized using Digoxigenin-dUTP with mouse tail-tip DNA.

### Reverse-transcription PCR (RT–PCR)

Total RNA was isolated from 15 embryos using Isogen (Nippon Gene) according to the manufacturer's instructions. Rabbit α-globin mRNA (100 pg) was added as an external control before RNA isolation. Reverse transcription was performed using the PrimeScript RT-PCR kit (Takara) according to the manufacturer's protocol. PCR reactions were performed in a thermal cycler (iCycler; Bio-Rad) using Ex Taq DNA polymerase (Takara), with the cDNA derived from one embryo as a template. The sequences of a common primer pair for Flag-H3.1 and Flag-H3.2 were 5′-ATGACGACGATAAGGGAGGA-3′ and 5′-CTCGGTCGACTTCTGGTAGC-3′. The primer pair sequences for cyclin A2 were 5′-GAGGTGGGAGAAGAATATAA-3′ and 5′-ACTAGGTGCTCCATTCTCAG-3′. The PCR conditions were: 95°C for 2 min, followed by 30 cycles of 95°C for 30 sec, 60°C for 30 sec and 72°C for 30 sec. The PCR products were then subjected to electrophoresis. Real-time quantitative PCR was performed with the Smart Cycler System (Takara) using the following primer pairs: H3.1: 5′-TGCAGGAGGCCTGTGA-3′ and 5′-TGGATGTCCTTGGGCATG-3′, H3.2: 5′-TGCAGGAGGCGAGCGA-3′ and 5′-TGGATGTCCTTGGGCATG-3′, rabbit α-globin: 5′-GCAGCCACGGTGGCGAGTAT-3′ and 5′-ATTTCAAGCTCCTGTCCCAC-3′. The PCR conditions were: 95°C for 10 sec, followed by 40 cycles of 95°C for 5 sec and 60°C for 20 sec.

### Immunoblotting

A total of 150 two-cell embryos that had been injected with Flag-H3.1 or Flag-H3.2 mRNA before fertilization were incubated at 95°C for 5 min in 2× SDS sample buffer and stored at −20°C. The proteins were separated by SDS-PAGE on a 10% polyacrylamide gel and electrically transferred to polyvinylidene fluoride membranes (Millipore) in transfer buffer (25 mM Tris-HCl, 192 mM glycine, 20% methanol). The membranes were blocked for 60 min in Tris-buffered saline (TBS) containing 0.1% Tween-20 (Wako) and 2% ECL Advance blocking agent (Amersham). They were washed in TBS/0.1% Tween-20 and then incubated with anti-Flag M2 antibody diluted 1∶1000 in TBS/2% ECL Advance blocking agent overnight at 4°C. The membranes were washed once and then incubated with horseradish peroxidase-conjugated anti-mouse IgG (Cell Signaling) for 1 h at room temperature. The membranes were washed in TBS/0.1% Tween-20, and the immunoreactivity was visualized using an ECL Advance Detection Kit (Amersham) and detected using a Luminescent Image Analyzer (LAS-1000; Fujifilm). The membranes were reprobed with α-tubulin (Sigma-Aldrich, 1∶4000) as a loading control.

### RNA interference

CAF-1 p150- and EGFP-specific siRNA duplexes were chemically synthesized and purified by Invitrogen. The siRNA sequences were designed using the Block-iT™ RNAi Designer program (Invitrogen). Two different siRNAs targeting p150 were prepared: 5′-CCAACUGCACGAGUUCCGACUUGAA-3′ and 5′-GAUGCCCAACUUGGAGGAUGCUGUU-3′. The sequence of the EGFP siRNA was 5′-CCACUACCUGAGCACCCAGUCCGCC-3′. The siRNAs were diluted to 20 µM in nuclease-free water and injected into the cytoplasm of one-cell embryos.

## Supporting Information

Figure S1 DNA replication dependence of Flag-H3 variant deposition in somatic cells. The deposition of Flag-H3 variants into the nuclei of NIH 3T3 cells treated without (–Aphi) or with aphidicolin (+Aphi) was analyzed by immunostaining with anti-Flag antibody. The DNA was counterstained with propidium iodide. The cells were transfected with each Flag-H3 variant expression vector, treated with aphidicolin 12 h post-transfection, and then fixed 48 h post-transfection. The percentage of transfected cells showing Flag-H3 variant deposition in the nuclei is shown in the upper right corner of each panel. Scale bar, 100 µm.
(TIF)

Figure S2 Disappearance of H3.3 from the maternal genome in parthenogenetically activated oocytes. Fully grown oocytes were microinjected with Flag-H3.3 mRNA in the presence of IBMX, which inhibits meiotic maturation. IBMX was washed out 5 h later, and the oocytes were matured to MII-stage oocytes. Next, the oocytes were parthenogenetically activated through treatment with 10 mM SrCl_2_ in Ca^2+^-free KSOM for 1 h. The activated oocytes were incubated for an additional 3 h in KSOM and then immunostained with anti-Flag antibodies.
(TIF)

Figure S3 Deposition of Flag-H3.3 is maintained after the M phase of the first embryonic cell cycle. One-cell embryos at the G2 phase were microinjected with Flag-H3.3 mRNA, cultured, and then collected for immunostaining 16 or 22 h after insemination, at which points the embryos were at the one-cell M phase (1-cell M) or the two-cell G1 phase (2-cell G1), respectively. Because aphidicolin treatment to inhibit DNA synthesis was initiated 16 h after insemination, none of the two-cell embryos entered the S phase. The DNA was stained with propidium iodide. Flag-H3.3 was detected both in the mitotic chromosomes of one-cell embryos and in the nuclei of two-cell embryos. Scale bar, 10 µm.
(TIF)

Figure S4 Validation of the differences in nuclear incorporation patterns between H3.1 and H3.2 during the early preimplantation stage. Flag-H3.1 and -H3.2 mRNAs with identical 5′-untranslated regions (UTRs) containing Kozak sequences, 3′-UTRs, and poly(A) sites were synthesized using the pcDNA3.1-poly(A)83 plasmid [36]. mRNA was injected into MII-stage oocytes, which were fertilized *in vitro* 2 h later. (A) Oocytes that had been injected with the indicated concentrations of mRNA were fertilized and collected at the one- or two-cell stage (11 and 28 h after fertilization, respectively) for immunostaining. (B) Immunoblotting with an anti-panH3 antibody (Abcam: ab1791) showed that exogenous H3.1 and H3.2 (synthesized following the injection of 50 ng/μl mRNA) had accumulated in amounts comparable to endogenous H3 before the first round of DNA replication (5 h after fertilization). A total of 180 embryos were loaded per lane. (C) Analysis of the incorporation of EGFP-H3.1 and -H3.2 into zygotic chromatin. As EGFP fusion proteins somehow localize to the nucleoplasm (data not shown), one-cell embryos at M phase were collected for immunostaining in order to detect the incorporation of histones into chromatin.
(TIF)

Figure S5 Deposition of Flag-H3.2 and Flag-H3.3 in DNA replication-dependent and -independent manners in one-cell stage embryos. (A) The experimental scheme is shown. The timing of DNA replication at the one-cell stage was determined by BrdU pulse labeling for 30 min at hourly intervals (data not shown). DNA replication was detected in whole nuclei from 5 to 7 h post insemination. Eight hours after insemination, DNA replication was detected only in partial regions of nuclei. To analyze H3 variant incorporation at the G1, S, and G2 phases, Flag-H3 variant mRNAs were microinjected 2 h prior to fertilization and 4 and 8 h post insemination, respectively, and then each embryo was collected for immunocytochemistry at 4, 9, and 13 h post insemination. Aphidicolin treatment was provided from 5 h after fertilization. Scale bar, 10 µm. (B) Upper pronucleus is of maternal origin (m), and lower pronucleus is of paternal origin (p). The one-cell embryos were fixed for immunostaining 4 h (G1), 9 h (S), and 13 h (G2) after fertilization. Incorporation of Flag-H3.2 and Flag-H3.3 at the S phase was prevented by aphidicolin (S+Aphi), whereas no decrease in Flag-H3.3 incorporation at the G2 phase in the presence of aphidicolin (G2+Aphi) was observed. Scale bar, 10 µm.
(TIF)

Figure S6 Chromatin reorganization during preimplantation development. (A) Nuclear DNA in oocytes and preimplantation embryos was stained with PI. In the nuclei of one- and two-cell embryos, chromatin is mostly decondensed, and heterochromatin domains, which are characterized by DNA-dense foci, are confined to the peripheries of nucleoli. However, between the two-cell and four-cell stages, heterochromatin domains separate from the peripheries of nucleoli and localize at discrete foci in the nucleoplasm. The heterochromatin foci increase and become distinct as the embryos develop into the blastocyst stage. Arrowheads indicate representative heterochromatic regions. Scale bar, 20 µm. (B) DNA FISH for major satellites, which represent the predominant heterochromatic region, shows that DNA-dense foci were co-localized with major satellites and that the localization of major satellites was dynamically changed during preimplantation development.
(TIF)

Figure S7 Co-localization of Flag-H3 variants with major satellites at the two-cell and blastocyst stages. Immuno-DNA-FISH analysis showed that Flag-H3.2 and Flag-H3.3 were co-localized with major satellites in the nucleolar peripheral regions (arrows) at the two-cell stage. At the blastocyst stage, Flag-H3.1 and Flag-H3.2 were co-localized with major satellites (arrows), but Flag-H3.3 was hardly co-localized with them.
(TIF)

Figure S8 Heterochromatin formation is significantly reduced in sip150-treated embryos. Heat-map analysis showed that heterochromatin regions were reduced significantly in sip150-treated embryos compared with siEGFP-treated embryos. The red area shows DNA-dense heterochromatin regions stained with DAPI. We quantified the red area in the nucleus of sip150-treated embryos and siEGFP-treated embryos, revealing that DNA-dense regions decreased significantly in sip150-treated embryos (*t*-test, *P*<0.005). Scale bar, 20 µm.
(TIF)

Figure S9 Salt extractability and post-translational modification of N-terminal tagged histones. To confirm that N-terminally Flag tagged histone H3.1 form proper nucleosomes and are modified correctly, ES cells stably expressing N-terminal Flag-tagged H3.1 or H3.3 were prepared. (A) Solubility at various concentrations of NaCl. ES cells stably expressing N-terminal Flag-tagged H3.1 were lysed in an isotonic buffer with 0-2 M NaCl and pelleted. Proteins from whole cells (total), the pellet (pellet) and the supernatant (sup) were analyzed by immunobotting with an anti-panH3 antibody (Abcam: ab1791). Tagged and untagged histones showed similar extraction profiles, indicating that N-terminal tagged histones are tightly bound to chromatin like endogenous ones. (B) Patterns of histone modifications. Nucleosomes were prepared by micrococcal nuclease treatment of the nuclei from N-terminal Flag-tagged H3.1 and H3.3 expressing cells followed by immnoprecipitation with anti-Flag antibody. On the left, we confirmed that mononucleosomes immunoprecipitated with anti-Flag (IP) contained DNA fragments of the appropriate size (∼150 bp), which was also observed in pre-immunoprecipitation sample (total). The immunoprecipitated samples were analyzed by immunoblotting with the antibodies against H3K4me3 (Millipore: 07-473) and H3K9me3 (Abcam: ab8898). Flag-H3.3 was enriched in K4me3 whereas Flag-H3.1 was enriched in K9me3.
(TIF)
