# Supplementary figures and images for: Dynamic Replacement of Histone H3 Variants Reprograms Epigenetic Marks in Early Mouse Embryos
Source: PLoS Genet. 2011 Oct 6;7(10):e1002279. doi: 10.1371/journal.pgen.1002279 (PMC3188537; doi:10.1371/journal.pgen.1002279)

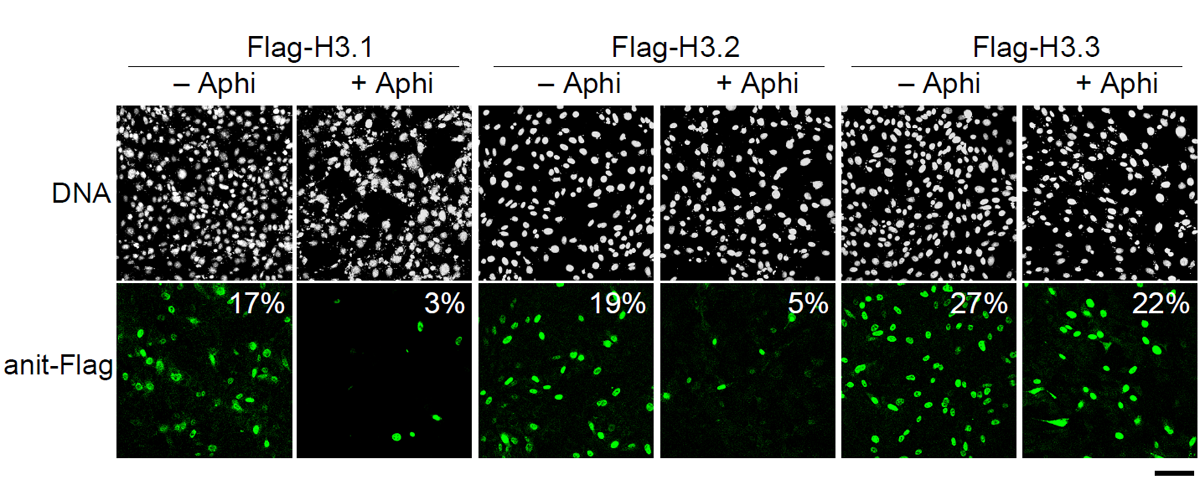

Supplement: Figure S1 — DNA replication dependence of Flag-H3 variant deposition in somatic cells. The deposition of Flag-H3 variants into the nuclei of NIH 3T3 cells treated without (–Aphi) or with aphidicolin (+Aphi) was analyzed by immunostaining with anti-Flag antibody. The DNA was counterstained with propidium iodide. The cells were transfected with each Flag-H3 variant expression vector, treated with aphidicolin 12 h post-transfection, and then fixed 48 h post-transfection. The percentage of transfected cells showing Flag-H3 variant deposition in the nuclei is shown in the upper right corner of each panel. Scale bar, 100 µm. (TIF) [file pgen.1002279.s001.tif]

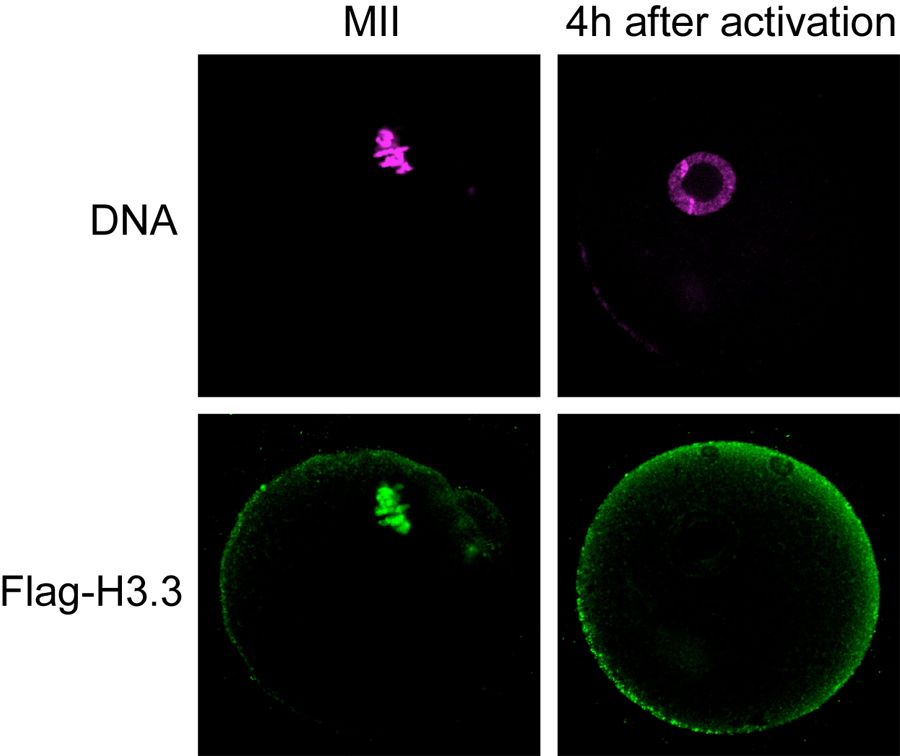

Supplement: Figure S2 — Disappearance of H3.3 from the maternal genome in parthenogenetically activated oocytes. Fully grown oocytes were microinjected with Flag-H3.3 mRNA in the presence of IBMX, which inhibits meiotic maturation. IBMX was washed out 5 h later, and the oocytes were matured to MII-stage oocytes. Next, the oocytes were parthenogenetically activated through treatment with 10 mM SrCl2 in Ca2+-free KSOM for 1 h. The activated oocytes were incubated for an additional 3 h in KSOM and then immunostained with anti-Flag antibodies. (TIF) [file pgen.1002279.s002.tif]

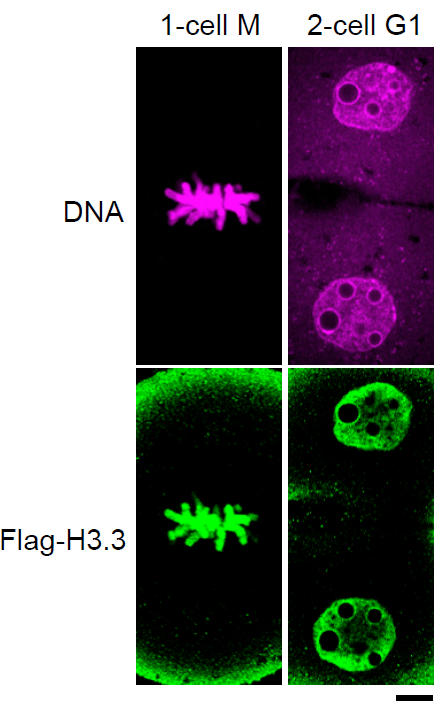

Supplement: Figure S3 — Deposition of Flag-H3.3 is maintained after the M phase of the first embryonic cell cycle. One-cell embryos at the G2 phase were microinjected with Flag-H3.3 mRNA, cultured, and then collected for immunostaining 16 or 22 h after insemination, at which points the embryos were at the one-cell M phase (1-cell M) or the two-cell G1 phase (2-cell G1), respectively. Because aphidicolin treatment to inhibit DNA synthesis was initiated 16 h after insemination, none of the two-cell embryos entered the S phase. The DNA was stained with propidium iodide. Flag-H3.3 was detected both in the mitotic chromosomes of one-cell embryos and in the nuclei of two-cell embryos. Scale bar, 10 µm. (TIF) [file pgen.1002279.s003.tif]

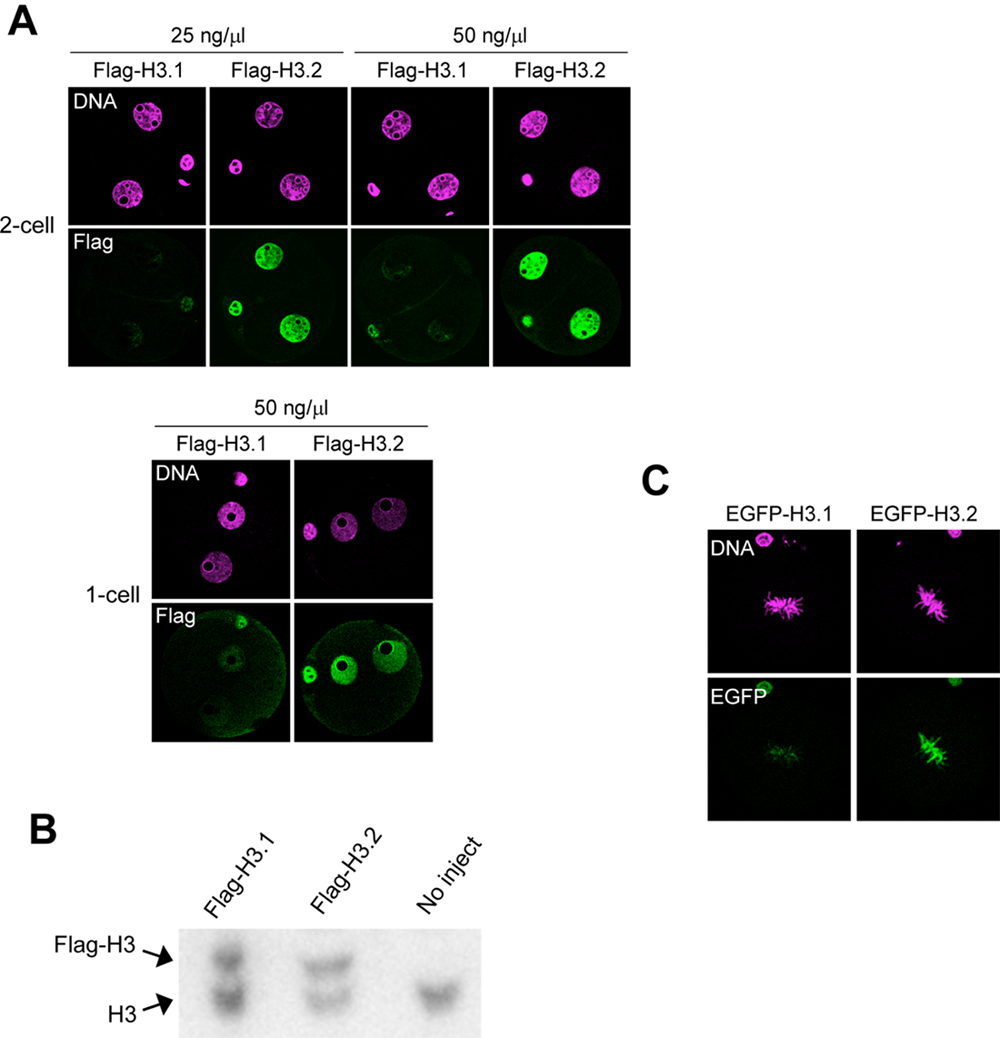

Supplement: Figure S4 — Validation of the differences in nuclear incorporation patterns between H3.1 and H3.2 during the early preimplantation stage. Flag-H3.1 and -H3.2 mRNAs with identical 5′-untranslated regions (UTRs) containing Kozak sequences, 3′-UTRs, and poly(A) sites were synthesized using the pcDNA3.1-poly(A)83 plasmid [36]. mRNA was injected into MII-stage oocytes, which were fertilized in vitro 2 h later. (A) Oocytes that had been injected with the indicated concentrations of mRNA were fertilized and collected at the one- or two-cell stage (11 and 28 h after fertilization, respectively) for immunostaining. (B) Immunoblotting with an anti-panH3 antibody (Abcam: ab1791) showed that exogenous H3.1 and H3.2 (synthesized following the injection of 50 ng/μl mRNA) had accumulated in amounts comparable to endogenous H3 before the first round of DNA replication (5 h after fertilization). A total of 180 embryos were loaded per lane. (C) Analysis of the incorporation of EGFP-H3.1 and -H3.2 into zygotic chromatin. As EGFP fusion proteins somehow localize to the nucleoplasm (data not shown), one-cell embryos at M phase were collected for immunostaining in order to detect the incorporation of histones into chromatin. (TIF) [file pgen.1002279.s004.tif]

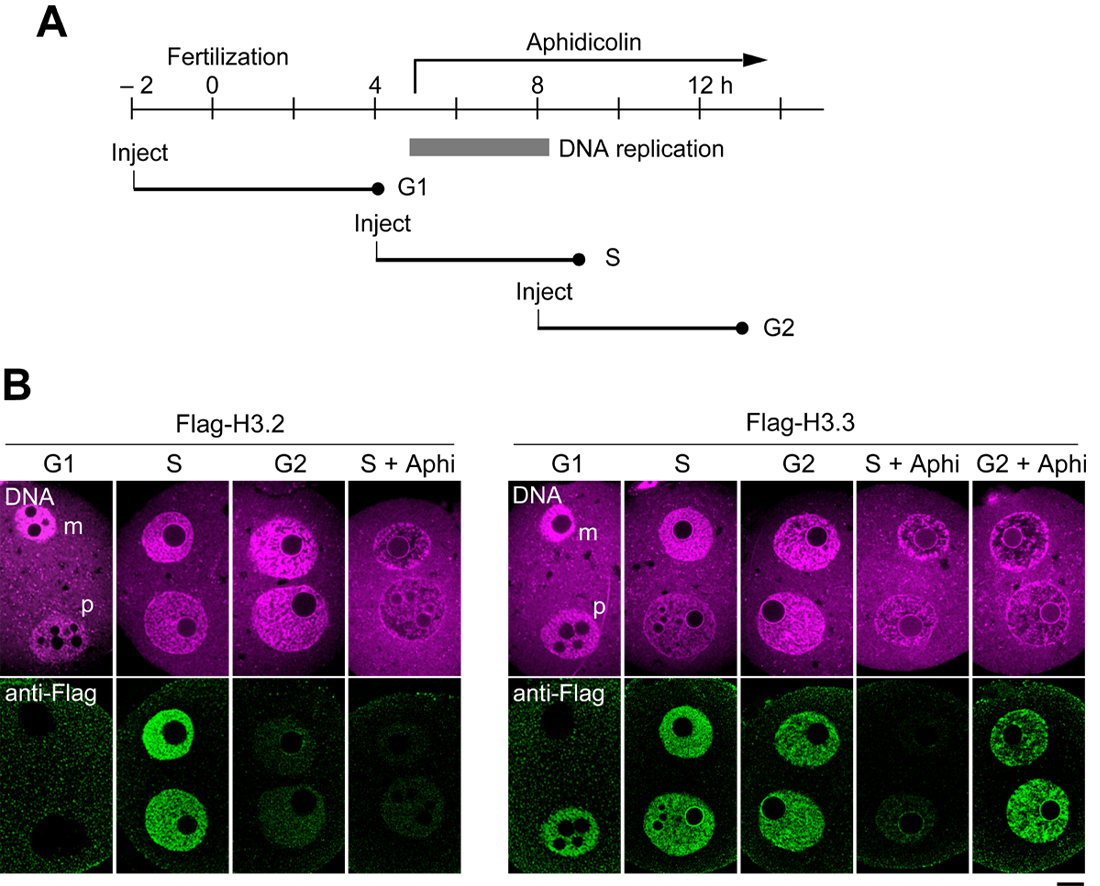

Supplement: Figure S5 — Deposition of Flag-H3.2 and Flag-H3.3 in DNA replication-dependent and -independent manners in one-cell stage embryos. (A) The experimental scheme is shown. The timing of DNA replication at the one-cell stage was determined by BrdU pulse labeling for 30 min at hourly intervals (data not shown). DNA replication was detected in whole nuclei from 5 to 7 h post insemination. Eight hours after insemination, DNA replication was detected only in partial regions of nuclei. To analyze H3 variant incorporation at the G1, S, and G2 phases, Flag-H3 variant mRNAs were microinjected 2 h prior to fertilization and 4 and 8 h post insemination, respectively, and then each embryo was collected for immunocytochemistry at 4, 9, and 13 h post insemination. Aphidicolin treatment was provided from 5 h after fertilization. Scale bar, 10 µm. (B) Upper pronucleus is of maternal origin (m), and lower pronucleus is of paternal origin (p). The one-cell embryos were fixed for immunostaining 4 h (G1), 9 h (S), and 13 h (G2) after fertilization. Incorporation of Flag-H3.2 and Flag-H3.3 at the S phase was prevented by aphidicolin (S+Aphi), whereas no decrease in Flag-H3.3 incorporation at the G2 phase in the presence of aphidicolin (G2+Aphi) was observed. Scale bar, 10 µm. (TIF) [file pgen.1002279.s005.tif]

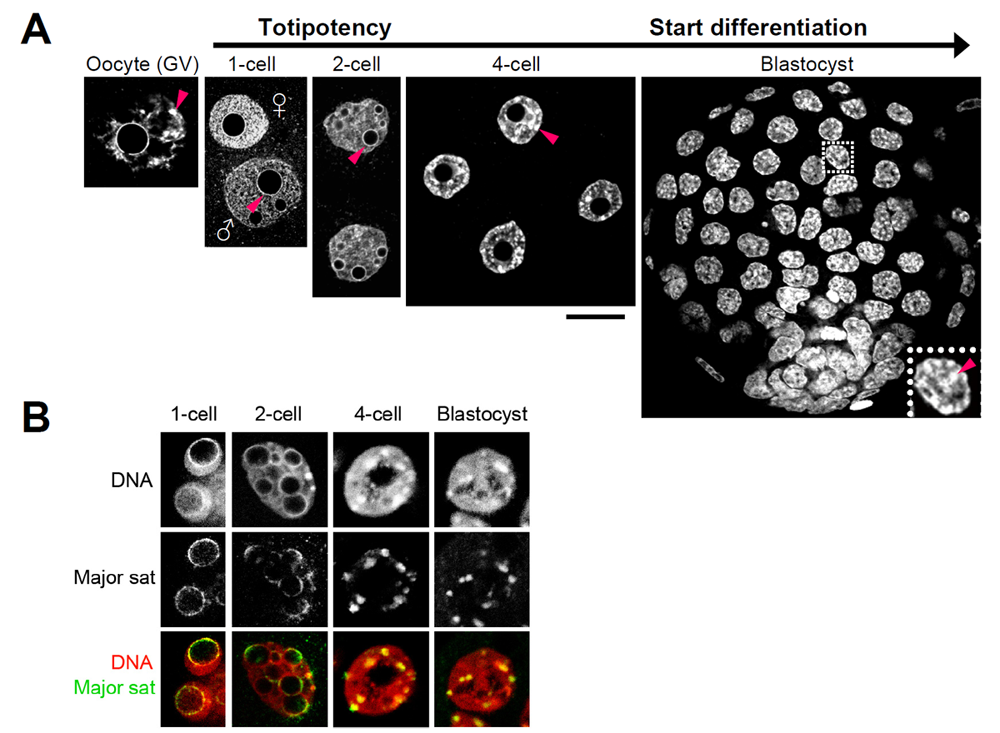

Supplement: Figure S6 — Chromatin reorganization during preimplantation development. (A) Nuclear DNA in oocytes and preimplantation embryos was stained with PI. In the nuclei of one- and two-cell embryos, chromatin is mostly decondensed, and heterochromatin domains, which are characterized by DNA-dense foci, are confined to the peripheries of nucleoli. However, between the two-cell and four-cell stages, heterochromatin domains separate from the peripheries of nucleoli and localize at discrete foci in the nucleoplasm. The heterochromatin foci increase and become distinct as the embryos develop into the blastocyst stage. Arrowheads indicate representative heterochromatic regions. Scale bar, 20 µm. (B) DNA FISH for major satellites, which represent the predominant heterochromatic region, shows that DNA-dense foci were co-localized with major satellites and that the localization of major satellites was dynamically changed during preimplantation development. (TIF) [file pgen.1002279.s006.tif]

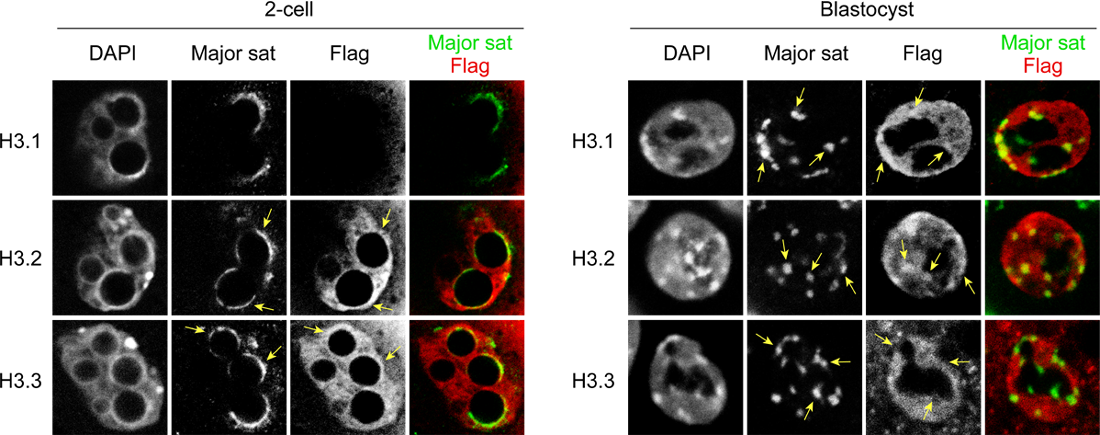

Supplement: Figure S7 — Co-localization of Flag-H3 variants with major satellites at the two-cell and blastocyst stages. Immuno-DNA-FISH analysis showed that Flag-H3.2 and Flag-H3.3 were co-localized with major satellites in the nucleolar peripheral regions (arrows) at the two-cell stage. At the blastocyst stage, Flag-H3.1 and Flag-H3.2 were co-localized with major satellites (arrows), but Flag-H3.3 was hardly co-localized with them. (TIF) [file pgen.1002279.s007.tif]

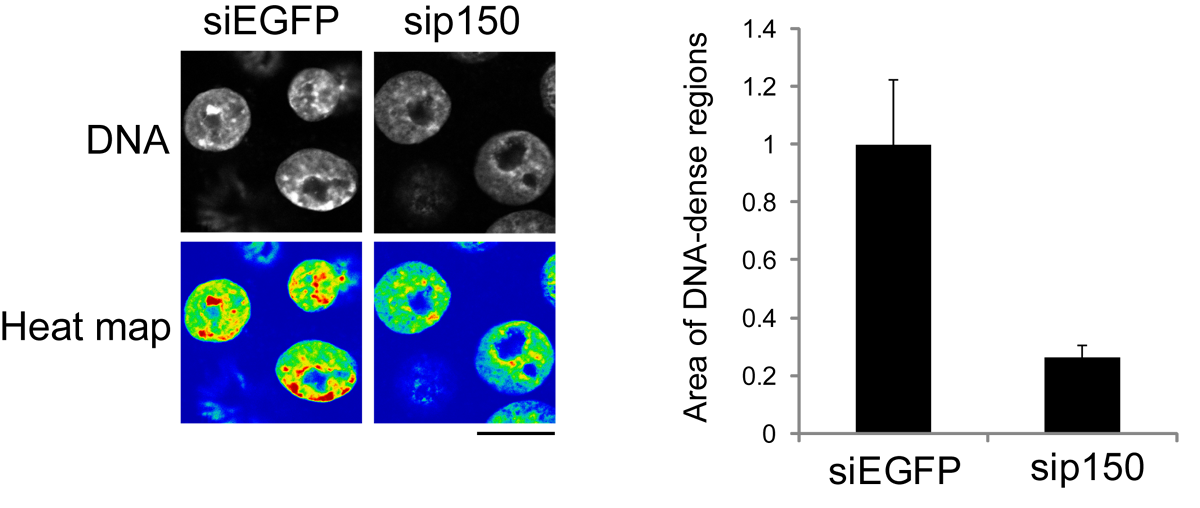

Supplement: Figure S8 — Heterochromatin formation is significantly reduced in sip150-treated embryos. Heat-map analysis showed that heterochromatin regions were reduced significantly in sip150-treated embryos compared with siEGFP-treated embryos. The red area shows DNA-dense heterochromatin regions stained with DAPI. We quantified the red area in the nucleus of sip150-treated embryos and siEGFP-treated embryos, revealing that DNA-dense regions decreased significantly in sip150-treated embryos (t-test, P<0.005). Scale bar, 20 µm. (TIF) [file pgen.1002279.s008.tif]

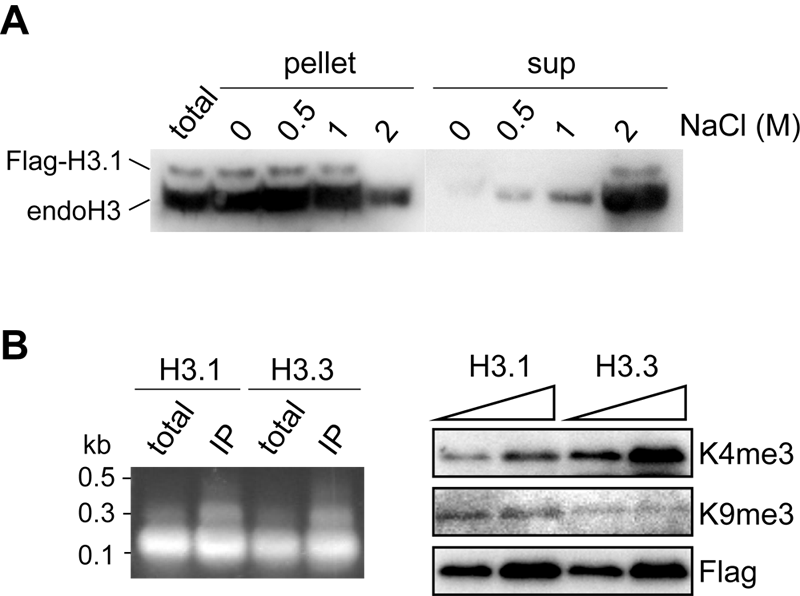

Supplement: Figure S9 — Salt extractability and post-translational modification of N-terminal tagged histones. To confirm that N-terminally Flag tagged histone H3.1 form proper nucleosomes and are modified correctly, ES cells stably expressing N-terminal Flag-tagged H3.1 or H3.3 were prepared. (A) Solubility at various concentrations of NaCl. ES cells stably expressing N-terminal Flag-tagged H3.1 were lysed in an isotonic buffer with 0-2 M NaCl and pelleted. Proteins from whole cells (total), the pellet (pellet) and the supernatant (sup) were analyzed by immunobotting with an anti-panH3 antibody (Abcam: ab1791). Tagged and untagged histones showed similar extraction profiles, indicating that N-terminal tagged histones are tightly bound to chromatin like endogenous ones. (B) Patterns of histone modifications. Nucleosomes were prepared by micrococcal nuclease treatment of the nuclei from N-terminal Flag-tagged H3.1 and H3.3 expressing cells followed by immnoprecipitation with anti-Flag antibody. On the left, we confirmed that mononucleosomes immunoprecipitated with anti-Flag (IP) contained DNA fragments of the appropriate size (∼150 bp), which was also observed in pre-immunoprecipitation sample (total). The immunoprecipitated samples were analyzed by immunoblotting with the antibodies against H3K4me3 (Millipore: 07-473) and H3K9me3 (Abcam: ab8898). Flag-H3.3 was enriched in K4me3 whereas Flag-H3.1 was enriched in K9me3. (TIF) [file pgen.1002279.s009.tif]
